# Kindness: Definitions and a pilot study for the development of a kindness scale in healthcare

**DOI:** 10.1371/journal.pone.0288766

**Published:** 2023-07-19

**Authors:** Austin B. Hake, Stephen G. Post

**Affiliations:** Department of Family, Center for Medical Humanities, Compassionate Care & Bioethics, Population & Preventative Medicine, Renaissance School of Medicine at Stony Brook University, Stony Brook, New York, United States of America; Faculty of Medicine, Saint-Joseph University, LEBANON

## Abstract

**Background:**

Empathy and compassion currently receive the most attention in healthcare with respect to the medical humanities and while these skills are important for any clinician to learn, they are complex and can be daunting to healthcare trainees when first encountered. Kindness is a simple, time-sensitive behavior not yet well characterized in the healthcare setting. With this study, we aim to clearly define it as well as investigate a few common examples of kindness that might be used to create a scale for use in the healthcare setting.

**Methods:**

A literature search was performed to rigorously define kindness. A kindness scale based on this definition was then compiled and administered to 45 patients across three outpatient clinical settings to evaluate the association between several actions and the patient’s perception of kindness.

**Results:**

Kind actions are small, take little effort, and are short in duration to their intended effect. We define kindness as an action that benefits another, as perceived by the recipient of the kind action. The results from our clinical study indicate several actions such as greeting the patient with a smile, asking questions about the patient’s daily life, listening carefully, and appearing interested in the patient have a moderate strength correlation to a perception of kindness. The physician being perceived as kind also had a weak-moderate strength correlation to the patient subjectively reporting improvement after their visit.

**Conclusions:**

Definitions in the medical humanities are important as they guide the scales used to measure them. This article defines kindness and describes some examples of its manifestation in the healthcare setting. Our study indicates that performing kind actions may improve a patient’s subjective perception of their care, however, future studies are needed to evaluate whether this benefit extends to health outcomes as has been demonstrated for skills such as empathy and good communication.

“Constant kindness can accomplish much. As the sun makes ice melt, kindness causes misunderstanding, mistrust, and hostility to evaporate.”

~*Albert Schweitzer*

## Introduction

Kindness is a largely unacknowledged skill in patient care [1]. While bioethics and humanities courses in medical education have become ever more prevalent, empathy and compassion have been the two traits that have found firm footing in such curricula [2–4]. There has been a robust production of effective courses aimed at teaching these virtues [5,6], but learning empathy and compassion can be challenging, the best methods are still debated [7–9], and there continue to be reports of medical students’ empathy diminishing as they are exposed to the realities of the clinical setting [10]. The debate about the best method to teach these virtues may persist because definitions of empathy and compassion vary and the validated scales used to measure these behaviors differ [11–14]. We say this not to diminish the importance of empathy or compassion, but to demonstrate their complexity and describe the challenge of measuring positive virtues whose definitions are not clear and fully agreed on [7,9].

Kindness has always been a core component of medicine from the *Corpus Hippocraticum* mention of “*philia*,*”* to the earliest journal publications and the development of the term “good bedside manner” [15,16]. As the rhetoric of describing humanistic characteristics of clinicians has shifted from bedside manner to empathy and compassion, it leaves out the small, simple, reliable actions of kindness that are essential to good bedside manner. These sorts of actions, like sitting down and looking a patient in the eye, noticing a patient’s bedside decorations, or just listening, have powerful effects and may help engender trust with the patient [17–19]. Elevating kindness in medical education should not be an onerous task. Everyone is familiar with the term, and its meaning has been understood by all long before they were introduced to empathy and compassion. Instead of suggesting a new initiative or humanistic trait to combat the detached nature of contemporary medicine, a refocusing and reaffirming of simple, core kindness seems an easier route to take. As medical trainees are guided from routine care to compassion in their professional education, kindness may be an easier place to begin a process of practice and dialogue [20].

The aim of this article, in the relatively sparse landscape of clinical kindness research, is threefold. One, we aim to set forth a rigorously defined explanation of kindness. Two, we aim to use that definition to compile a list of potential scale items about kindness and design a pilot cross-sectional clinical study to correlate them to a patient’s subjective perception of physician kindness. Three, we aim to use this pilot study to evaluate the correlation between a perception of kindness and the patient’s subjective perception of care and trust in their physician.

## Methods

To search for existing conceptions of kindness, a literature search was conducted through PubMed and Embase for articles about kindness, its definition, and how it has been measured. Additionally, prominent non-fiction books on kindness and the patient experience were reviewed [17–19,21–23]. Finally, reference novels and sites were also reviewed [24–28].

The survey used in our pilot study was generated by drawing scale items from the three existing scales on kindness, which focus on non-clinical contexts [29–31]. We also examined validated scales on empathy [13,14], compassion [11,12], and other interpersonal traits [32–35]. Items were included in the proposed kindness scale, at the discretion of the authors, if the item matched our proposed definition and if the item was applicable and measurable in the healthcare setting (S1 Table). The final proposed kindness scale is shown in Table 1 items 1–6. These items were correlated to item 7 to investigate their correlation to a perception of kindness in accordance with aim two. Items 8 and 9 were included to measure the patient’s subjective perception of their care after being seen by the physician and item 10 was included to assess trust in the physician in accordance with aim three.

**Table 1:** Kindness survey items.

1. The doctor greeted me with a smile
2. The doctor made eye contact with me while talking to me
3. The doctor asked about what was happening in my daily life
4. The doctor dismissed my concerns too easily
5. The doctor listened carefully to me
6. The doctor seemed interested in me
7. The doctor was kind
8. I felt better after talking with my doctor
9. The doctor made me feel at ease
10. I trusted the doctor

Our pilot cross-sectional clinical survey study was approved under Stony Brook Hospital IRB#2020–00786. Physicians were enrolled to recruit patients and their informed consent was obtained. Physicians were blinded to the purpose of the study so as to not affect their encounter with the patient and were only told that the study was to measure the “interaction between patient and physician.” Adult English-speaking participants were recruited by their physicians from suburban outpatient clinics. After a patient had a visit from a participating physician and the patient had agreed to participate in the study, author Hake, A.B. would enter the room and obtain full informed consent by discussion and consent form. The patient would then be provided with the survey to complete, with the reassurance that all responses were anonymous and there would be no possible way to connect their responses back to them. All items on the scale were graded on a 5-tier Likert scale from “strongly disagree” to “strongly agree.”

Data was collected on Qualtrics XM. Final data was transferred to an encrypted and password-protected server where statistical analysis was performed using SAS 9.4. Wilcoxon signed-rank, Kruskal-Wallis, and Spearman’s rank correlation nonparametric statistical tests were used for comparative data analysis.

## Results

A total of 45 patients were recruited between January 2021 and January 2022. Table 2 summarizes the demographics of the participants and the distribution of clinics the survey was administered in. There were no significant differences in physician rating for any of the scale values across age, demographics, or clinic specialty (S2 Table).

**Table 2:** Demographics of study.

Age, mean (SD)	63 (18)
Gender	
Female, n (%)	30 (66)
Male, n (%)	15 (33)
Race/Ethnicity	
Hispanic, n (%)	5 (11)
Asian, n (%)	2 (4)
White, n (%)	38 (85)
Specialty Clinic	
Primary Care, n (%)	34 (76)
Obstetrics/Gynecology, n (%)	8 (18)
Neurology, n (%)	3 (6)

Table 3 displays the items of the kindness scale with their mean response values. Nearly all participants rated their physicians as kind, resulting in an average response score of 4.98 (SD 0.15), where 5 would be all patients responding with “strongly agree.” All other scale items listed in Table 3 showed a similar trend except for item 4, which was reverse worded, thus an average value close to 1 would follow the scale trend for this item. Examination of the responses for item 4 also indicated a range of 1–5, suggesting there might have been confusion when answering the question. The Cronbach Alpha score for the scale was 0.65, which rose to 0.74 after item 4 was removed. A value of >0.70 indicates high internal consistency of the scale [36].

**Table 3:** Survey response values.

Survey item:	Mean (SD)
1. The doctor greeted me with a smile	4.87 (0.63)
2. The doctor made eye contact with me while talking to me	5.00 (0.00)
3. The doctor asked about what was happening in my daily life	4.84 (0.42)
4. The doctor dismissed my concerns too easily	1.56 (1.29)
5. The doctor listened carefully to me	4.91 (0.29)
6. The doctor seemed interested in me	4.98 (0.15)
7. The doctor was kind	4.98 (0.15)
8. I felt better after talking with my doctor	4.78 (0.60)
9. The doctor made me feel at ease	4.76 (0.65)
10. I trusted the doctor	5.00 (0.00)

Graded on a 5-tier Likert scale from “strongly disagree” (1) to “strongly agree” (5).

Table 4 describes the correlation analysis of the scale. Two scale items (2 and 10), received unanimous scores of 5, precluding them from the correlation analysis. Except for item 4, our proposed measures of kindness compiled from existing scales (items 1–6 in Table 1) had a significant moderate strength correlation with a patient’s perception that their physician was kind (item 7). The scale item with the strongest correlation with a physician being kind was item 6, “the physician seeming interested in the patient.” Scale items that evaluated the patient’s subjective perception of improvement after being seen by their physician (items 8 and 9 in Table 1) also demonstrated a significant mild-moderate strength correlation with a physician being perceived as kind.

**Table 4:** Spearman correlations between the different scale items.

Survey item:	Item 1	Item 3	Item 4	Item 5	Item 6	Item 7	Item 8	Item 9
Item 1	1	0.40**	-0.07	0.22	0.55***	0.55***	0.13	0.11
Item 3		1	0.04	0.55***	0.37**	0.37**	0.80***	0.57***
Item 4			1	-0.21	-0.26	-0.26	0.05	-0.08
Item 5				1	0.48***	0.48***	0.53***	0.52***
Item 6					1	1***	0.34*	0.32*
Item 7						1	0.34*	0.32*
Item 8							1	0.72***
Item 9								1

Item 2 (eye contact) and 10 (trust) could not be included as there were no variance in their values. <0.3 is a weak correlation, 0.3–0.7 is a moderate correlation, >0.7 is a strong correlation.

*p<0.05

**p<0.01

***p<0.001.

## Discussion

### Definition of kindness

The definition of kindness varies in the literature. Some authors claim it stems from a feeling of love [21], others claim it stems from empathy [25], and some liken it to altruism in that kindness is a “helping behavior” [24]. One can see the similarities in these ideas, but love is too intense an emotion, apart from a broad “love of humanity.” Empathy requires resonating with another’s emotions [22]. Altruism and generosity require there be some cost to the altruistic person, and as actions, they need not involve the feeling or attitude of kindness [37]. Canter *et al*. describe a slightly more accurate representation of kindness as a “gesture motivated by genuine warm feeling for others” [29]. Here, we see the key aspects of kindness: It is an action (mental or physical), that has a “warm” component, “directed toward fostering… well-being or flourishing” [38]. Considered externally, kindness consists of an action, followed by some sort of benefit to the person the action is directed towards.

A final consideration to complete the definition is whether the benefit must merely be intended, as Canter *et al*. and others [28] suggest, or must be recognized as such from the recipient’s perspective. Various articles about kindness in other contexts seem to conclude that kindness is perceived by the recipient [31,39]. It is the result, not the intention of kindness that counts. Anecdotally, this conclusion agrees with our conception of kindness. Imagining someone who gifted food to their coworker that contained an ingredient the coworker was allergic to, we would say they were *trying* to be kind, but did not successfully perform a kind act.

A few philosophical and technical explorations of kindness include how in terms of the awareness the recipient has about the kind action, all they must encounter is some benefit, they do not need to be aware that their benefit was derived from an action by someone else. A clinician that walks into a sleeping patient’s room and chooses to return later performs a kind act, but the patient is oblivious as to how they were allowed to rest longer. Kindness also has both a scale and temporality associated with it. Kind acts are relatively simple, they generally do not involve major effort or time, and the benefit is usually felt quite proximately to the action [29]. Kindness additionally modifies actions in that for any particular action, there are both kind and unkind ways to perform that action. Finally, kindness can be a trait in that as someone becomes known to perform kind actions, they become known as a kind person.

In sum, kindness is an action that benefits another, as perceived by the person the kind act is directed towards.

### Types of kindness

We affirm that two types of kindness exist. One type of kindness, distinguished by Harding as *Everyday Kindness* [18], involves actions performed without any special insight into how the other person will react to the action. The other type of kindness, *Informed Kindness*, involves actions where something is known about the other person’s perceptions of what a kind act to them would be.

Everyday Kindness describes most of the experiences we perceive as kind, such as picking up a pen for someone who dropped it, opening a door for someone, listening attentively, or offering a smile and ‘good morning’ as you pass colleagues going into work. A close synonym might be “friendly” or “nice.” There is no guarantee that these actions will be perceived as kind by the other person, but based on shared culture and socialization, we have a fairly good idea of what might be considered kind by other people. An important note on Everyday Kindness is that as these actions are generated based on socialization, they may not translate well across cultures.

Informed Kindness describes actions where additional information about the other person is employed so that there is a higher certainty that the action will be perceived as kind. A close synonym might be someone performing a “thoughtful” action. This could take the form of knowing someone does not want a door opened for them and thus not doing so, knowing a friend’s favorite food and purchasing it for them, knowing someone cannot hear on their right side and purposely speaking to their left, or knowing someone has a preferred name different from their given name. These actions do not necessarily involve empathy, just factual information about a person’s preferences. It is possible that Informed Kindness acts could progress to involve empathy, for example, noticing that someone is upset and resonating with their demeanor in such a way that you sense they would prefer to go about the day as normal instead of being asked: “What’s wrong?” This level of emotional connection, however, almost seems to supersede kindness.

We propose only these two types of kindness, however, it is possible that kindness in other settings could take on new forms. In one of the types of kindness Canter *et al*. describe, “Principled Proaction,” they note it contained several examples of altruism and generosity [29]. They also describe a common attribute of kindness called “Core Kindness” that contains three items, one of them being “I like to make other people feel happy,” which seems quite close to the definition of kindness we arrived at in this manuscript. It may be possible that herein we are describing this “Core Kindness,” and that kindness, when applied to certain situations takes on other forms. These other forms could be altruism or generosity when selflessly donating money or time, and compassion when relieving suffering.

### Kindness in healthcare

“Small acts of kindness can personalise care and often take little time to perform. Getting the patient a glass of water, helping them with their slippers, getting them their glasses or hearing aid, adjusting a pillow or their bed sheets, acknowledging a photograph, greetings card, or flowers—these behaviours convey a powerful message, indicating that the person is worthy of such attention.”

~Harvey Chochinov

Our pilot study demonstrates that actions such as smiling, asking about a patient’s daily life, listening carefully, and seeming interested in the patient are associated with a physician being perceived as kind. Additional kind actions that could apply in all patient encounters include sitting down at the level of the patient, asking the patient if they need anything or would like any further clarification at the end of the visit, using responsive body language like nodding, speaking in a slow and warm tone, or asking permission to perform physical exam maneuvers. As in Dr. Chochinov’s quote above [40], there are also many contextual examples of kindness in the hospital such as re-adjusting the bedding to how it was before the physical exam was performed, cleaning off ultrasound gel when finished, adjusting nasal cannulas so that they are not uncomfortable, or exhibiting care when peeling off tape or EKG adhesive stickers, among many others.

Despite our results indicating physicians are already mostly kind, a recent study by Dignity Health found that 64% of patients in their 1,000-patient sample experienced unkind treatment such as rudeness and poor listening skills [41]. Furthermore, the same study found that a strong majority of respondents listed kind care as *more important* than wait time, cost of care, or length of travel. Clearly, patients desire kind care and in some cases, they do not receive it.

Medical education interventions aimed at improving humanistic skills like empathy are often implemented as an educational course and it seems likely kindness could be taught in a similar fashion [42]. What may differentiate kindness as more easily teachable is that it is more amenable to being described as a quick checklist to follow when interacting with patients. Whereas instructions to improve empathy might include suggestions like imagining what it is like in the patient’s shoes or identifying emotions [13], improving kindness could be as simple as instructing the physician to greet the patient with a smile or ask one question about their patient’s daily life unrelated to their medical complaint.

### Health implications of kind care

A large body of research has been developing over the past decades describing the health outcome benefits of non-pharmacological interventions like empathy, compassion, and communication skills. A meta-analysis of 13 randomized control trials found the effect size of a good patient-physician relationship on health outcomes such as blood pressure control, asthma severity, osteoarthritis pain, weight loss, and re-consultation rate was 0.11 [43]. For context, the effect size of Aspirin for secondary prevention of all major vascular events is 0.12 [44], which is an undisputed cornerstone of guideline-directed vascular disease care [45]. While a good patient-physician relationship is not necessarily the same as kind care, many of the measures used in the studies within Kelley *et al*. included actions such as listening carefully, allowing the patient to express their story, not interrupting, and using encouraging body language, which do seem like acts of kindness [46,47].

Another meta-analysis of over 100 correlational studies on good communication with patients found that the odds of patient adherence was 2.16 times higher if the physician communicated effectively. It also concluded that over 180 million office visits a year could result in better patient adherence if their doctors communicated better [48]. Again, good communication is not the same as kindness, but they do share many features. A final study demonstrated that patients who rated their physician as having a perfect CARE score, an empathy scale with many items of kindness included, had decreased cold severity and duration [49]. Humanistic skills seem to improve patient health outcomes and the volume of research supporting this claim continues to grow [18,22,50–55].

The behaviors measured in these studies are not the same as kindness, but the similarities in the scales used and our results demonstrating a weak to moderate strength correlation between acts of kindness and a patient’s subjective perception of improvement are intriguing. Future work evaluating health outcomes using a specific kindness scale is needed to qualify kindness in this list of humanistic behaviors that can provide health outcome benefits.

## Limitations and future questions

There are a few limitations of this pilot study, namely the sample size, which while meeting statistical significance for many of our questions, was not large enough for specialty comparison or for a factor analysis of the scale items. The inherent biases present in many survey studies such as extreme response bias, acquiescence bias, and demand characteristic bias affected our study, and the unanimous “strongly agree” responses to scale items 2 and 10 precluded us from performing correlation analysis on them. While only one physician refused to refer patients for this study, another consideration may be that the type of doctors amenable to partaking in a study like this may be more kind to begin with.

This paper aimed to create a scale to measure kindness from an empirical approach. Thus, while on a definitional basis we have distinguished kindness from other humanistic behaviors like empathy, the scale was not procedurally confirmed to measure kindness as distinct from those other behaviors. Future work might seek to compare this kindness scale to a scale such as the Jefferson scale of empathy to investigate whether patients make this sort of humanistic differentiation in physicians from their perspective.

Questions for future efforts include whether it is indeed the kind act and/or is it the sentiment of feeling cared for that is responsible for generating good health outcomes in a patient. As an example, is it the act of smiling at a patient that produces the effect of kindness, or is it that in smiling at a patient, the patient perceives that the physician cares for them, and that resultant feeling in the patient is what drives the benefits of kindness. Lastly, a future project would use this kindness scale to develop a study evaluating the correlation between kind acts and health outcomes to determine whether kindness follows other humanistic skills in its ability to modulate physical health benefits.

## Conclusion

Educating medical trainees about empathy and compassion has been an excellent addition to many medical curricula, but these concepts are complex and can be daunting to the student when first encountering them. We highlighted kindness in this article as a less challenging method to improve a physician’s bedside manner, with examples from our pilot study of acts of kindness that patients associate with physicians being kinder. Definitions are essential in bioethics, especially if scales are to be built around that definition, thus great effort was taken to define kindness in a rigorous manner. *The resulting definition describes kindness as an act that benefits another*, *as perceived by the recipient of the kind action*. In the healthcare setting, studies about good communication, empathy, and compassion have shown an improvement in health outcomes, and some of that improvement might be due to kindness. Further studies using a specific kindness scale are still needed, however, to investigate this claim. Kindness may improve a patient’s subjective perception of their care, is beneficial for the provider [56], requires little time, and should be highlighted more in medical settings as a component of good bedside manner.

## Supporting information

S1 Table Kindness scale items and original scale reference language. (DOCX)

S2 Table Statistical analysis for demographic differences among kindness survey items. (DOCX)

S1 Data Survey data. (CSV)
