# Supplementary material for: Kindness: Definitions and a pilot study for the development of a kindness scale in healthcare
Source: PLoS One. 2023 Jul 19;18(7):e0288766. doi: 10.1371/journal.pone.0288766 (PMC10355430; doi:10.1371/journal.pone.0288766)
Supplement: S1 Table — (DOCX) [file pone.0288766.s001.docx]

**Table S1. Kindness scale item and original scale reference language.**

| Item | Original reference text | Reference |
| --- | --- | --- |
| 1 | “I smile at strangers” | [29] |
| 2 | “When speaking with the patient, try to be seated at a comfortable distance for conversation, at the patient’s eye level when possible” | [40] |
| 3 | “Asks about what is happening in my daily life” | [13] |
| 4 | “My doctor dismisses my concerns too easily” | [34] |
| 5 | “How often did doctors listen carefully to you?” | [35] |
| 6 | “I feel that my doctor is interested in me” | [32] |
