# Supplementary material for: Kindness: Definitions and a pilot study for the development of a kindness scale in healthcare
Source: PLoS One. 2023 Jul 19;18(7):e0288766. doi: 10.1371/journal.pone.0288766 (PMC10355430; doi:10.1371/journal.pone.0288766)
Supplement: S2 Table — (DOCX) [file pone.0288766.s002.docx]

**Table S2A. Statistical difference between genders for each scale item**

| Item | Wilcoxon 2-sided Z test p-value |
| --- | --- |
| 1 | 0.9778 |
| 2 | 1.0000 |
| 3 | 0.9512 |
| 4 | 0.2058 |
| 5 | 0.7325 |
| 6 | 0.5093 |
| 7 | 0.5093 |
| 8 | 0.3078 |
| 9 | 0.5796 |
| 10 | 1.0000 |

**Table S2B. Statistical difference between race/ethnicities for each scale item**

|  | Kruskal-Wallis test | | |
| --- | --- | --- | --- |
| Item | Chi-Square | DF | Pr > ChiSq |
| 1 | 0.5783 | 2 | 0.7489 |
| 2 | 0.0000 | 2 | 1.0000 |
| 3 | 1.2434 | 2 | 0.5370 |
| 4 | 9.9895 | 2 | 0.0068* |
| 5 | 0.7908 | 2 | 0.6734 |
| 6 | 0.1842 | 2 | 0.9120 |
| 7 | 0.1842 | 2 | 0.9120 |
| 8 | 1.2413 | 2 | 0.5376 |
| 9 | 1.4819 | 2 | 0.4767 |
| 10 | 0.0000 | 2 | 1.0000 |

*This statistically significant value is likely solely due to human oversight when encountering this reverse-worded item, which disproportionally occurred in one racial/ethnic group in the dataset

**Table S2C. Statistical difference between clinics for each scale item**

|  | Kruskal-Wallis test | | |
| --- | --- | --- | --- |
| Item | Chi-Square | DF | Pr > ChiSq |
| 1 | 0.6245 | 2 | 0.7318 |
| 2 | 0.0000 | 2 | 1.0000 |
| 3 | 1.5266 | 2 | 0.4661 |
| 4 | 2.5760 | 2 | 0.2758 |
| 5 | 0.4123 | 2 | 0.8137 |
| 6 | 4.6250 | 2 | 0.0990 |
| 7 | 4.6250 | 2 | 0.0990 |
| 8 | 1.2508 | 2 | 0.5351 |
| 9 | 1.2114 | 2 | 0.5457 |
| 10 | 0.0000 | 2 | 1.0000 |

**Table S2D. Statistical difference among age for each scale item**

| Item | Spearman correlation test p-value |
| --- | --- |
| 1 | 0.6695 |
| 2 | N/A |
| 3 | 0.8463 |
| 4 | 0.1470 |
| 5 | 0.3143 |
| 6 | 0.0902 |
| 7 | 0.0902 |
| 8 | 0.7260 |
| 9 | 0.7902 |
| 10 | N/A |
